# Nanoemulsion-based delivery system for enhanced oral bioavailability and Caco-2 cell monolayers permeability of berberine hydrochloride

**DOI:** 10.1080/10717544.2017.1410257

**Published:** 2017-12-01

**Authors:** Yong-Jiang Li, Xiong-Bin Hu, Xiu-Ling Lu, De-Hua Liao, Tian-Tian Tang, Jun-Yong Wu, Da-Xiong Xiang

**Affiliations:** a Department of Pharmacy, The Second Xiangya Hospital, Central South University, Changsha, Hunan, China;; b Key Laboratory of Traditional Chinese Medicine Preparations of Hunan Province, the Second Xiangya Hospital, Central South University, Changsha, Hunan, China;; c Hunan Cancer Hospital, Changsha, Hunan, China;; d Institute of Clinical Pharmacy, Central South University, Changsha, Hunan, China

**Keywords:** Berberine hydrochloride, bioavailability, Caco-2 cells, transport, nanoemulsion

## Abstract

Berberine hydrochloride (BBH) has a variety of pharmacological activities such as antitumor, antimicrobial, anti-inflammation, and reduce irritable bowel syndrome. However, poor stability and low oral bioavailability limited its usage. Herein, an oil-in-water nanoemulsion system of BBH was developed to improve its stability and oral bioavailability. The pseudoternary phase diagrams were constructed for the determination of composition of various nanoemulsions. The nanoemulsions of BBH composed of Labrafil M 1944 CS (oil phase), RH-40 (surfactant), glycerin (co-surfactant), and water (aqueous phase). The O/W nanoemulsion of BBH showed a relative bioavailability of 440.40% compared with unencapsulated BBH and was stable in our 6-month stability study. Further, there was a significant increase in intestinal permeability of BBH as assessed by Caco-2 cell monolayers and a significant reduction in efflux of BBH by the multidrug efflux pump P-glycoprotein. This study confirmed that the nanoemulsion formulation could be used as an alternative oral formulation of BBH to improve its stability, oral bioavailability and permeability.

## Introduction

Berberine hydrochloride (BBH) is an isoquinoline alkaloids distributed in various natural herbs. BBH has a variety of pharmacological activities such as antitumor (James et al., 2011; Tan et al., 2011; Cheng et al., 2015), antimicrobial (Zhang et al., 2010; Sun et al., 2014; Wen et al., 2016), anti-inflammation (Lee et al., 2007; Wen et al., 2016), and reduce irritable bowel syndrome(Chen et al., 2015; Habtemariam, 2016). However, oral administration of BBH exhibited low bioavailability and poor stability (Tan et al., 2011; Liu et al., 2014) mainly due to its hydrophobic properties resulted from the chemical structure, which contains two methoxy groups and a quaternary ammonium cation. Besides, the cationic group in the structure shows high affinity to the multidrug efflux pump P-glycoprotein (P-gp) in gastrointestinal tract (Zhang & Cui, 2008; Qiu et al., 2009; Najar et al., 2010). In addition, the apparent oil-water partition coefficient of BBH determined in our preliminary study (lg*P_app_* = −1.08) indicated low membrane permeability (Lu et al., 2011). Therefore, it is of great importance to develop a novel drug delivery system to improve the solubility and bioavailability of BBH (Shen et al., 2010; Tan et al., 2011).

Nanoemulsion is a thermodynamically stable system and transparent dispersions of oil and water with the droplet size less than 100 nm stabilized by an interfacial film of surfactant molecules. In recent years, nanoemulsions has become a promising approach for the delivery of poorly water-soluble drugs to improve oral bioavailability as well as to modify drug release characteristics (Aboalnaja et al., 2016; Akhtar et al., 2016; Khani et al., 2016). The mechanisms for enhanced absorption of nanoemulsions are very complex, involving improved solubility of drug molecules, formation of mixed micelles, opening of tight junctions, and improved lymphatic delivery (Qi et al., 2011; Aboalnaja et al., 2016). The aim of this study was to develop a nanoemulsion delivery system of BBH to improve its stability, oral bioavailability, and to evaluate its *in vitro* membrane permeability using Caco-2 cell model (Balimane et al., 2004; Piazzini et al., 2017).

## Materials and methods

### Materials

Caster oil, Labrafil M 1944 CS, Labrasol, RH-40, Tween-20, Labrafac Lipophile wl 1349, PEG-400, 1,2-propanediol, glycerin, 1,3-butanediol, dehydrated alcohol, ammonium dihydrogen phosphate, and BBH raw powder were purchased from Xian Xiaocao Botanical Development Co. (Xi’an, China; Batch No. XC100913, purity ≥98.2%), BBH tablets was produced by Northeast General Pharmaceutical Factory (Shenyang, China; Batch no. 100132), BBH (standard), and *p*-dimethylamino benzaldehyde (standard) were purchased from National Institutes for Food and Drug Control (China; Batch nos. 110713-200911 and 0791-9102, respectively); acetonitrile and methanol were of HPLC grade and supplied from TEDIA Co. (Fairfield, OH); 0.1% – trypsin and l-glutamine were purchased from GIBCO Co. (Carlsbad, CA); fetal bovine serum (FBS) was purchased from TIAN JIN HAO YANG BIOLOGICAL MANUFACTURE CO (Tianjin, China); Dulbecco’s modified Eagle’s medium, 0.01 mol L^−1^, phosphate buffer salt (PBS), and D-hank’s solution were supplied by the Experiment Center of The Second Xiangya Hospital of Central South University (Hunan, China); 100 U mL^−1^ penicillin–streptomycin solution was purchase from Beyotime Institute of Biotechnology (Jiangsu, China). Distilled water was produced by the Pharmaceutical Preparation Room of The Second Xiangya Hospital of Central South University (Hunan, China). All other chemicals were of the analytic grade.

### Methods

#### Solubility study

The solubility of BBH in various oils, surfactants, and co-surfactants was determined by adding excess amount of BBH into 10 mL of each vehicle in the centrifugal tube, then vortexed in a Constant Temperature Vibrator (Thermo Co., Waltham, MA) at 37.0 °C for 24 h. After the equilibrium was achieved, the mixture was centrifuged at 3000 rpm for 15 min, and each supernatant was filtered through a 0.45 μm filter. Then the concentration of BBH in the supernatant was determined by high-performance liquid chromatography (HPLC) (HP1200, Agilent, Palo Alto, CA).

#### Pseudo-ternary phase diagrams study

Based on the results of solubility study (Table S1), Labrafil M 1944 CS and Caster oil were selected as the candidates of oil phase, Tween 20, RH-40 and Labrasol were selected as the candidates of surfactants, PEG-400, 1,2-propanediol, glycerin, 1,3-butanediol and ethanol were selected as the candidates of co-surfactants. Three different weight ratios (*K*_m_ of 3:1, 2:1, and 1:1) of surfactant to co-surfactant were investigated; also, five different weight ratios (*K*_m_ of 9:1, 8:2, 7:3, 6:4, and 5:5) of the surfactant/co-surfactant to the oil were investigated (Mehta et al., 2009).

Distilled water was progressively titrated to multi-component mixtures of oil, surfactant, and co-surfactant at ambient temperature to construct pseudoternary phase diagrams by visually observing the mixture after gentle magnetic stirring. After equilibration, the mixtures were visually assessed as nanoemulsion, crude emulsion, or gel. Based on the above results, the appropriate combination of oil/surfactant/co-surfactant/water was selected for the further experiments. The electrical conductivity was measured to determine the titration end-point and the formulation of nanoemulsion (Xiang et al., 2010).

#### Preparation of BBH nanoemulsion

Based on the results of construction of pseudoternary phase diagrams, we formulated the BBH nanoemulsion by Labrafil M 1944 CS/RH-40/glycerin/aqueous phase =0.50:3.39:1.13:13.50 (w/w/w/w) (Figure S1(A)). BBH was first dissolved in co-surfactant and then oil and surfactant were slowly added into the mixture with gentle stirring. After a transparent preparation was obtained, the formulated nanoemulsion was stored under ambient temperature in sealed glass vial. In order to determine the maximum loading content of BBH in the nanoemulsion formulation, excess amount of BBH was dissolved into the prepared blank nanoemulsion for 1 h. Excess BBH was removed by centrifugation at 13,000 rpm for 15 min, then the maximum loading content of BBH in the saturated nanoemulsion was determined by HPLC.

#### Stability study

About 10 mL prepared BBH nanoemulsion was stored in a 15 mL clean glass vial under the ambient temperature for 6 months. The appearance, centrifugal stability, concentration of BBH, mean droplet size, and polydispersity index (PDI) were investigated at every 2 months to assess the physical stability of BBH nanoemulsion. The centrifugal stability was tested by a high-speed centrifuge (Hunan Saite Xiangyi Centrifugal Instrument Company, China), the nanoemulsion vehicles were centrifuged for 30 min at 13,000 rpm. The amount of BBH was determined by HPLC, and the particle size and PDI were determined by NANO-ZS90 Nano Particle Analyzer (Malvern, Britain).

#### HPLC analysis

The amount of BBH in various vehicles was determined by an Agilent 1200 HPLC system, equipped with an Agilent 1200 series UV–Vis DAD detector (Agilent Technologies, Palo Alto, CA). The chromatographic column was a reverse phase Agilent HC-C_8_ column (150 × 4.6 mm, 5 μm). The mobile phase consisted of acetonitrile, water and ammonium dihydrogen phosphate (25:75:0.1, v/v/v, pH value 2.80). The UV detector was set at *λ* = 345 nm, the flow rate was fixed at 1.0 mL min^−1^, and the column temperature was maintained at 25.0 °C.

#### Bioavailability study

Male Sprague–Dawley (SD) rats (225 ± 25 g) were purchased from the Animal Center of the Second Xiangya Hospital of Central South University (Hunan, China). Twelve SD rats were randomly divided into two groups: BBH nanoemulsion (Group A) and BBH suspension (Group B). The BBH suspension (10 mg mL^−1^) was made from BBH commercial tablets (100 mg/tablet, 5 tablets). All the rats ware administrated at the BBH dose of 62.5 mg kg^−1^.

Before the experiment, the rats were fasted overnight with free access to water. The rats were intragastrically administered BBH nanoemulsion and BBH suspension. Then, approximately 0.5 mL of blood sample was collected from caudal vein into the heparin-containing tubes at 10, 20, 30, 45, 60, 120, 180, 240, 360, 480, 720, and 1440 min after oral administration. Blood samples were centrifuged at 13,000 rpm for 5 min and plasma samples were stored frozen at −20 °C till HPLC analysis. The animal experimental procedures were approved by Intuitional Animal Ethical Committee and were in compliance with the guide of Institute of Laboratory Animal Resources Committee on Care and Use of Laboratory Animals.

About 200 μL aliquot of plasma sample was added into a new 1.5 mL centrifuge tube together with 50 μL of internal standard solution (*p*-dimethylaminobenzaldehyde, 1.0 μg mL^−1^) and 600 μL of methanol. Samples in all tubes were vortex-mixed for 3 min, and then centrifuged at 13,000 rpm for 10 min. The supernatants were then transferred into another tubes and evaporated to dryness at 35.0 °C using a Thermo Scientific Savant Explorer Speed Vac System (Thermo Fisher Scientific, Waltham, MA). The residue was then dissolved by 50 µL of methanol with vortex mixing for 5 min. The solution was centrifuged for 5 min at 13,000 rpm, and 20 µL of the supernatant was injected into the HPLC system.

#### *In vitro* permeability study across caco-2 cell monolayers

For the *in vitro* transport study of BBH nanoemulsion, the colonic adenocarcinoma cell line (Caco-2 cells) obtained from the Experiment Center of the Second Xiangya Hospital of Central South University (Hunan, China) were cultured in Dulbecco's modified eagle medium (DMEM) supplemented with 10% fetal bovine serum (FBS), 1% penicillin–streptomycin (100 U mL^−1^), and 1% l-glutamine at 37.0 °C in an atmosphere of 5% CO_2_. Then, cells were seeded onto the apical side of polycarbonate-coated Transwell-COL inserted in 12-well Transwell culture plate (Corning Costar Co., Corning, NY) at a concentration of 4 × 10^5^ cells/well. DMEM was added to apical (0.5 mL) and basolateral (1.5 mL) side, and was replaced every other day for the first week and daily thereafter. Cells were incubated for 18–22 d until the transepithelial electrical resistance (TEER) was measured by EVOM voltohmmeter (WPI Inc., Sarasota, FL). The integrity of Caco-2 monolayers was confirmed by TEER >300 Ω/cm^2^ (pH 7.2–7.4).

MTT assay was conducted to investigate the cytotoxic effects of BBH on Caco-2 cells. Caco-2 cells were harvested and seeded in standard 96-well microplates at a density of 1.3 × 10^4^ cells/well. They were subsequently treated with BBH nanoemulsion solution (diluted by DMEM containing 10% FBS) and BBH solution (dissolved in DMSO [<1%, v/v] and then diluted by DMEM containing 10% FBS). Both the solutions were at the BBH concentrations of 0.05, 0.075, 0.1, 0.5, 1, 2, and 4 mmol L^−1^ after filtering by microporous membrane (0.22 µm). The cells seeded on the 96-well microplates were treated by the solutions for 4 h with DMEM as the control. The cells were then incubated with MTT (20 μL) at 37 °C for another 4 h. Later, DMSO (100 μL) was added and incubated for 10 min at 37 °C. Optical density (OD) was observed at 490 nm using a Microplate Reader (Thermo Scientific, Waltham, MA). Cell viability was calculated as a percentage of DMEM control: cell viability (%) = (mean OD of experiment/mean OD of control) × 100%.

For the permeability study, cells were put into four groups: the control group (living cells only), BBH raw powder group, BBH nanoemulsion group, and BBH nanoemulsion + verapamil (Ver) group. The corresponding experimental solutions were prepared as follows: BBH raw powder group: BBH raw powder was dissolved in DMSO and diluted with D-Hank's balanced salt solution to the concentration of 100 μmol L^−1^ (DMSO <1%, v/v); BBH nanoemulsion group: BBH nanoemulsion was diluted with D-Hank's solution to the concentration of 100 μmol·L^−1^; BBH nanoemulsion + Ver group: the mix-solution contained 50 µmol L^−1^ Ver (He & Liu, 2002; Zhu & Liu, 2003; Bansal et al., 2009) and 100 μmol L^−1^ BBH nanoemulsion. The culture medium (DMEM) was replaced with pre-warmed (37.0 °C) transport medium consisting of D-Hank's solution. After the Caco-2 monolayers were equilibrated at 37.0 °C for 30 min, TEER values of monolayers were evaluated, and the D-Hank's solution was removed by aspiration. The permeability studies were performed in both directions (from apical to basolateral and from basolateral to apical). For the apical to basolateral (A-to-B) transport study, 0.5 mL of each experimental solution was added to apical side, while 1.5 mL D-Hank's solution was added to the basolateral side; for basolateral to apical (B-to-A) transport study, 1.5 mL of each experimental solution was added to basolateral side, while 0.5 mL D-Hank's solution was added to the apical side. And then they were cultured at 37.0 °C in an atmosphere of 5% CO_2_. Afterwards, 150 μL samples were collected from either the donor or receiving compartment at the predetermined time intervals of 15, 30, 45, 60, 90, 120, 150, and 180 min, and replenished with a corresponding volume of fresh D-Hank's solution. The samples were immediately stored below −20 °C for subsequent HPLC analysis. The mobile phase consisted of acetonitrile, water, and ammonium dihydrogen phosphate (25:75:0.1, v/v/v, pH 2.80). The UV detector was set at *λ* = 265 nm, the flow rate was fixed at 1.0 mL min^−1^ and the column temperature was maintained at 25.0 °C. The TEER values were monitored to ensure cell monolayers integrity.

*P_app_* was determined from the linear slope of the plot according to the following equation:

(1) $$P_{app}=\frac{dQ}{dt}\times \frac{1}{\left(A\times C_{0}\right)}$$

where *P_app_* is the apparent permeability coefficient (cm/s); d*Q*/d*t* is the steady state flux; *A* is the surface area of cell monolayer (cm^2^); *C*_0_ is the initial concentration of BBH in the apical (for A-to-B transport) or basolateral (for B-to-A transport) chamber.

The efflux rate (ER) was calculated according to the following equation:

(2) $$ER=\frac{P_{app\;ba}}{P_{app\;ab}}$$

where *P_app ba_* is the permeability co-efficient of B-to-A transport; *P_app ab_* is the permeability coefficient of A-to-B transport.

ER >1 indicates that P-gp transporter inhibited A-to-B transport (Ashiru-Oredope et al., 2011).

### Statistical analysis

Experiments were repeated three times and results were statistically expressed as mean ± standard deviation. All data analyses were performed using student’s t test at the significance level *α* = 0.05. All calculations were performed with the SPSS version 16.0 (SPSS, Chicago, IL).

## Results and discussion

### Solubility study

Results for solubility studies are presented in Table S1. Among the various vehicles tested, Labrafil M 1944 CS and Caster oil showed high solubilization capacity and were selected as the candidates of oil phase; Tween 20, ethoxylated hydrogenated castor oil-40 (RH-40) and Labrasol were selected as the candidates of surfactants and PEG-400, 1,2-propanediol, glycerin, 1,3-butanediol, and ethanol were selected as the candidates of co-surfactants for further experiments.

### Pseudo-ternary phase diagrams study

The pseudo-ternary phase diagrams of nanoemulsion composed of Labrafil M 1944 CS, RH-40, glycerin and water at various *K*_m_ are shown in Figure S1. The area of nanoemulsion formation was largest at *K*_m_ 3:1. The optimal formulation of surfactant/co-surfactant (S-CoS) to oil ratio was selected with the maximum amount of BBH at *K*_m_ 3:1. As a result, the S-CoS/oil ratio of 9:1 exhibited the maximum amount of BBH. Therefore, the best formulation of BBH was established: Labrafil M 1944 CS/RH-40/glycerin/water with *K*_m_ at 3:1 and S-CoS/oil at 9:1.

### Morphological characterization

BBH nanoemulsion was golden, clear, and transparent with good fluidity. The BBH nanoemulsion was spherical when observed under transmission electron microscopy (Figure S2).

### Stability of BBH nanoemulsion

BBH nanoemulsion was stored under the ambient temperature for 6 months. The appearance, centrifugal stability, amount of the drug, and droplet size were investigated at predetermined time (Table S2). Briefly, BBH nanoemulsion was centrifugally stable with no phase separation during the storage period. The amount of BBH and mean droplet size as well as the polydispersity index (PDI) were generally conform to requirements (Gutiérrez et al., 2008; Jaiswal et al., 2015), suggesting that BBH-loaded nanoemulsion was stable.

### Bioavailability study

**Figure 1.**
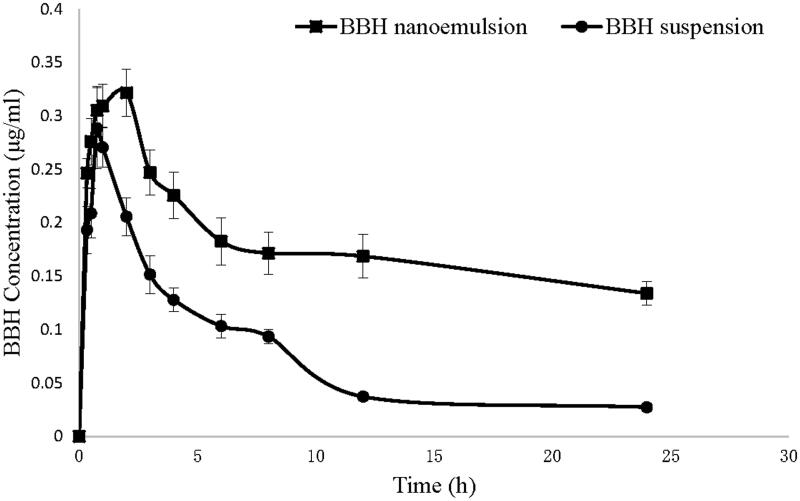
Plasma concentration profiles of BBH after oral administration of BBH nanoemulsion and BBH suspension in rats (*n* = 6).

Pharmacokinetic parameters of BBH nanoemulsion and BBH suspension were compared. Figure 1 shows the plasma concentration profiles of BBH after oral administration of both formulations in rats. The pharmacokinetic parameters are summarized in Table 1. The AUC_(0−∞)_ of BBH nanoemulsion was 4.4-fold than BBH suspension, indicating 440% relative bioavailability of BBH nanoemulsion compared with the suspension formulation. The *C*_max_ of BBH nanoemulsion was 1.6-fold than BBH suspension and the *T*_max_ was also significantly prolonged (4.3-fold) for BBH nanoemulsions compared with BBH suspension. The results suggested that the nanoemulsion could promote the absorption and sustain the release of BBH.

**Table 1.** Pharmacokinetics parameters after oral administration of BBH nanoemulsion and BBH suspension to rats (*n* = 6).

Parameters	BBH suspension	BBH nanoemulsion
AUC_(0–24)_ (mg/L h)	1.681 ± 0.558	4.963 ± 2.952
AUC_(0–∞)_ (mg/L h)	2.223 ± 0.810	9.790 ± 8.410
MRT_(0–24)_ (h)	10.009 ± 1.239	10.188 ± 2.391
MRT_(0–∞)_ (h)	17.944 ± 6.357	21.020 ± 10.950
*t*_1/2z_ (h)	9.761 ± 6.611	10.737 ± 7.027
*T*_max_ (h)	1. 141 ± 1.469	4.778 ± 5.645
*C*_max_ (mg L^−1^)	0.259 ± 0.179	0.404 ± 0.13
Relative bioavailability		440.40%

### *In vitro* permeability study across Caco-2 cell monolayers

**Figure 2.**
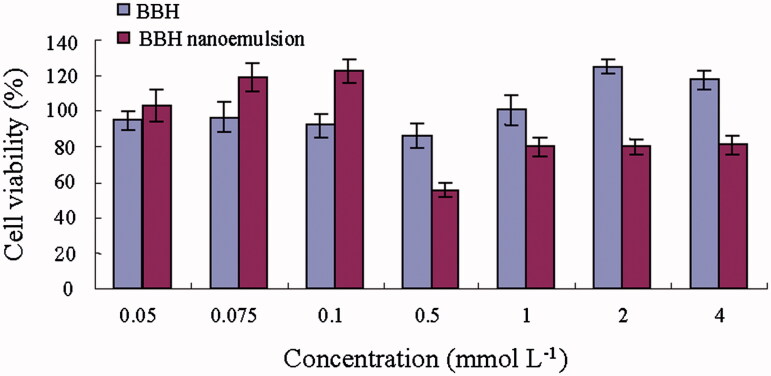
Effect of BBH solution and BBH nanoemulsion on Caco-2 cells viability as evaluated by the MTT assay after 4 h.

Methylthiazolyldiphenyl-tetrazolium bromide (MTT) assay was conducted to assess the concentration-dependent cytotoxic effects of BBH nanoemulsion and BBH solution (Figure 2). The BBH solution showed non-significant cytotoxicity (cell viability >90%) at the concentration between 0.05 and 4 mmol L^−1^, while BBH nanoemulsion showed non-significant cytotoxicity (cell viability >90%) at the concentration between 0.05 and 0.1 mmol L^−1^. Thus, the concentration of 100 μmol L^−1^ BBH solution and BBH nanoemulsion was selected for the in vitro permeability study.

**Figure 3.**
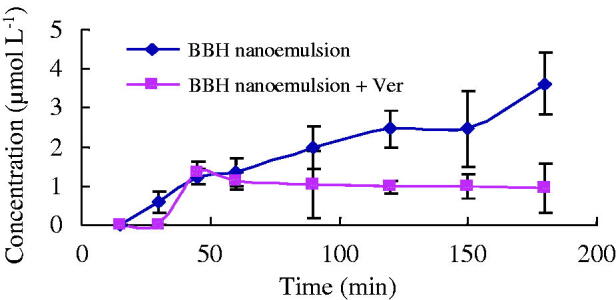
Absorption concentration of BBH nanoemulsion and BBH nanoemulsion + Ver from A→B in Caco-2 cell model (*n* = 3).

For the absorptive transport study (A-to-B), the absorptive concentration–time profiles of BBH raw powder was too low to be detectable by HPLC. The concentration of BBH in nanoemulsion formulation was increased with transport time, but when Ver was added, the concentration was lower than BBH nanoemulsion group and was not increased after 45 min (Figure 3), suggesting that Ver may have effect on the stability of BBH nanoemulsion.

**Figure 4.**
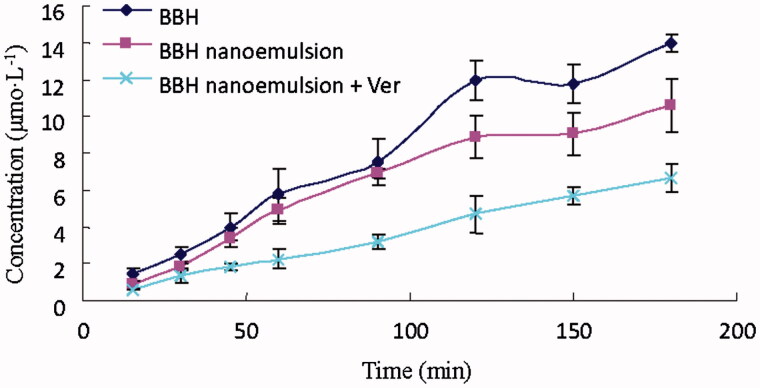
Efflux concentration of BBH, BBH nanoemulsion, and BBH nanoemulsion + Ver from B→A in Caco-2 cell model (*n* = 3).

For the efflux study (B-to-A), BBH raw powder showed highest concentration, BBH nanoemulsion showed moderate concentration, while BBH nanoemulsion + Ver showed the lowest concentration. The results indicated the presence of an efflux transporter (P-gp) for BBH (Figure 4). Besides, the apparent permeability coefficient (*P_app_*) (Bhushani et al., 2016) values of BBH from A-to-B transport and from B-to-A efflux were summarized (Table 2). Briefly, the efflux ratio of BBH nanoemulsion was 3.07 ± 0.34 highlighted the presence of an efflux transporter. The permeability coefficients of the BBH nanoemulsion and BBH nanoemulsion + Ver were significantly decreased compared with BBH. This indicated that nanoemulsion formulation may help to reduce the efflux of BBH in Caco-2 cell model and BBH was probably effluxed by P-gp as Ver, the P-gp inhibitor, significantly decreased the efflux of BBH (Figure 4).

**Table 2.** Apparent permeability coefficients (*P_app_*) and efflux ratio (ER) of BBH cross Caco-2 monolayer.

Group	*P_*app*_*(A→B) (10^-8^*cm s^*−1*^*)	*P_*app*_*(B→A) (10^-8^*cm s^*−1*^*)	*ER*
BBH	—	2.29 ± 0.11	—
BBH nanoemulsion	0.574 ± 0.18	1.76 ± 0.20**	3.07 ± 0.34
BBH nanoemulsion + Ver	0.185 ± 0.12*	1.07 ± 0.11#	5.78 ± 0.61^##^

* *p* < .05, compared with BBH nanoemulsion in A to B transport.

** *p* < .01, compared with BBH in B to A transport.

# *p* < .01, compared with BBH nanoemulsion in B to A transport.

## *p* < .01, compared with BBH nanoemulsion.

## Conclusions

In conclusion, the O/W nanoemulsion formulation composed of Labrafil M 1944 CS (oil), RH-40 (surfactant), and glycerin (co-surfactant) significantly improved the absorption of BBH and showed a 4.4-fold higher relative oral bioavailability in rats. In vitro permeability study across Caco-2 cell monolayers showed that nanoemulsion formulation could help to enhance the A-to-B transport and reduce the B-to-A efflux, and the absorption mechanism of BBH nanoemulsion in intestines may be passive transport as A-to-B, while P-gp is likely involved in the efflux of BBH nanoemulsion from B-to-A.

## Supplementary Material

IDRD_Li_et_al_Supplemental_Content.docx
