# Supplementary material for: Nanoemulsion-based delivery system for enhanced oral bioavailability and Caco-2 cell monolayers permeability of berberine hydrochloride
Source: Drug Deliv. 2017 Dec 1;24(1):1868–73. doi: 10.1080/10717544.2017.1410257 (PMC8240975; doi:10.1080/10717544.2017.1410257)
Supplement: IDRD_Li_et_al_Supplemental_Content.docx [file IDRD_A_1410257_SM2446.docx]

**Supplementary Information**

**Nanoemulsion based delivery system for** **enhanced oral bioavailability and Caco-2 cell monolayers permeability of berberine hydrochloride**

Yong-Jiang Li^1,2^, Xiong-Bin Hu^1,2^, Xiu-Ling Lu^1,2^, De-Hua Liao^1,2,3^, Tian-Tian Tang^1,4^, Jun-Yong Wu^1,2^, and Da-Xiong Xiang^1,2,3^

**Affiliation**

1 Department of Pharmacy, the Second Xiangya Hospital, Central South University, Changsha 410011, Hunan, China

2 Key Laboratory of Traditional Chinese Medicine Preparations of Hunan Province, the Second Xiangya Hospital, Central South University, Changsha 410011, Hunan, China

3 Hunan Cancer Hospital, Changsha 410006, Hunan, China

4 Institute of Mental Health, the Second Xiangya Hospital, Central South University, Changsha 410011, Hunan, China

**Correspondence**

Da-Xiong Xiang, PhD

Department of Pharmacy, the Second Xiangya Hospital, Central South University, Changsha 410011, Hunan, China. Tel: +86-0731-8529-2129

Email:xiangdaxiong@csu.edu.cn

**Supplementary Figures**


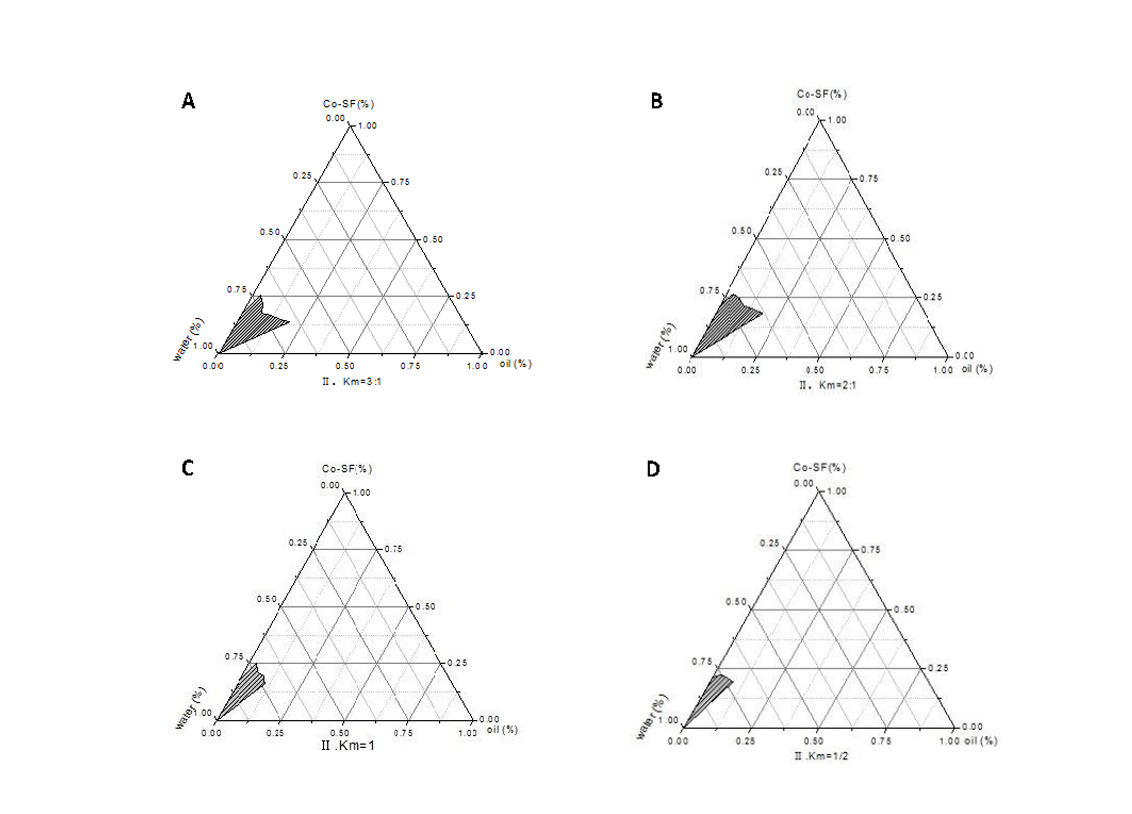


Figure S1. Pseudoternary phase diagrams indicating the efficient nanoemulsion region (A. Km=31, B. Km=21, C. Km=11, D. Km=12)


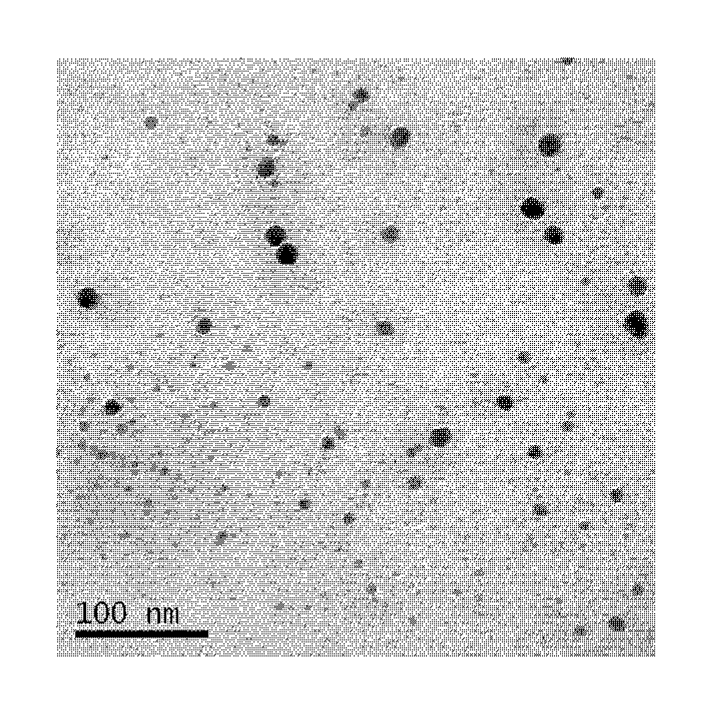


Figure S2. Transmission electron microscopy photograph of BBH nanoemulsion (×10000)

**Supplementary Tables**

**Table S1. Solubility of BBH in various vehicles at 37.0 ℃.**

|  | **Vehicles** | **Solubility (mg mL^-1^)** |
| --- | --- | --- |
| **Oil** | IPM | — |
|  | Caster oil | 0.2850 |
|  | Ethyl [oleate](app:ds:oleate) | — |
|  | Caprylic/capric triglyceride oil | — |
|  | Labrafil M 1944 CS | 0.1573 |
|  | Soybean oil | — |
| **Surfactant** | Tween-80 | 0.3506 |
|  | Tween-20 | 0.8771 |
|  | Labrasol | 3.0998 |
|  | Glycerol trioleate | 0.0165 |
|  | Cremophor EL-35 | 0.0260 |
|  | Cremophor RH-40 | 1.3143 |
| **Co-surfactant** | PEG-400 | 1.1836 |
|  | 1,2-propanediol | 3.7093 |
|  | Glycerin | 1.1115 |
|  | 1,3-butanedio | 8.8891 |
|  | Ethanol | 1.5516 |
|  | n-butanol | 0.0178 |

“—” undetectable concentration of BBH since it was lower than the detection limit.

**Table S2. Results of long-time stability test on appearance, centrifugal stability, droplet size and amount of the drug of BBH nanoemulsion.**

| **Formulation** | **Time**  **(month)** | **Appearance** | [**Centrifugal**](app:ds:centrifugal) [**stability**](app:ds:stability) | **Amount of BBH (mg·mL^-1^)** | **Mean droplet size (nm)** | **PDI** |
| --- | --- | --- | --- | --- | --- | --- |
| Labrafil M 1944 CS/ RH-40/Glycerin/water, Km=3, S-CoS/oil=9:1 | 0 | Transparent and golden | No separation | 2.39 | 17.46 | 0.059 |
|  | 1 | Transparent and golden | No separation | 2.32 | 19.37 | 0.277 |
|  | 3 | Transparent and golden | No separation | 2.21 | 18.62 | 0.268 |
|  | 6 | Transparent and golden | No separation | 2.27 | 19.12 | 0.218 |
